# Trend-Residual Dual Modeling for Detection of Outliers in Low-Cost GPS Trajectories

**DOI:** 10.3390/s16122036

**Published:** 2016-12-01

**Authors:** Xiaojian Chen, Tingting Cui, Jianhong Fu, Jianwei Peng, Jie Shan

**Affiliations:** 1 State Key Laboratory of Information Engineering in Surveying, Mapping and Remote Sensing, Wuhan University, Wuhan 430079, China; cxiaojian@whu.edu.cn (X.C.); cuitingting@whu.edu.cn (T.C.); 2 Collaborative Innovation Center of Geospatial Technology, Wuhan University, Wuhan 430079, China; 3 Research Center of Remote Sensing in Public Security, People’s Public Security University of China, Beijing 100038, China; 4 School of Remote Sensing and Information Engineering, Wuhan University, Wuhan 430079, China; fu_jianhong@whu.edu.cn (J.F.); pengjw@whu.edu.cn (J.P.); 5 Lyles School of Civil Engineering, Purdue University, West Lafayette, IN 47907, USA

**Keywords:** GPS trajectory, outlier detection, cubic smooth spline, time series, estimation

## Abstract

Low-cost GPS (receiver) has become a ubiquitous and integral part of our daily life. Despite noticeable advantages such as being cheap, small, light, and easy to use, its limited positioning accuracy devalues and hampers its wide applications for reliable mapping and analysis. Two conventional techniques to remove outliers in a GPS trajectory are thresholding and Kalman-based methods, which are difficult in selecting appropriate thresholds and modeling the trajectories. Moreover, they are insensitive to medium and small outliers, especially for low-sample-rate trajectories. This paper proposes a model-based GPS trajectory cleaner. Rather than examining speed and acceleration or assuming a pre-determined trajectory model, we first use cubic smooth spline to adaptively model the trend of the trajectory. The residuals, i.e., the differences between the trend and GPS measurements, are then further modeled by time series method. Outliers are detected by scoring the residuals at every GPS trajectory point. Comparing to the conventional procedures, the trend-residual dual modeling approach has the following features: (a) it is able to model trajectories and detect outliers adaptively; (b) only one critical value for outlier scores needs to be set; (c) it is able to robustly detect unapparent outliers; and (d) it is effective in cleaning outliers for GPS trajectories with low sample rates. Tests are carried out on three real-world GPS trajectories datasets. The evaluation demonstrates an average of 9.27 times better performance in outlier detection for GPS trajectories than thresholding and Kalman-based techniques.

## 1. Introduction

Low-cost, non-professional GPS (receiver) has become popular and grows in popularity at an ever increasing rate. Many devices in people’s daily life are equipped with a built-in, low-cost GPS, such as mobile phones, watches, and cars. Compared with professional GPS, low-cost GPS has advantages of easy use and portability. Although these GPS-enabled devices are found useful in online mapping [1], traffic management [2], and localization of places of interest [3], their accuracy is limited due to existence of outliers. Erroneous or missing GPS readings may be caused due to various reasons, such as obscured line of sight, device cold starts, and other satellite signal disruptions [4,5,6]. Such aberrant data with inherent inconsistency may lead to difficulties, mistakes, or even failures in subsequent processing such as route prediction [7] and trajectory clustering [8]. Moreover, outliers in a GPS trajectory hold back estimating some high-order trajectory properties, e.g., speed, heading, and acceleration, which impair the quality and efficiency of trajectory comprehension [9,10].

For further processing or analysis of GPS trajectories, data cleaning is a prerequisite for any value-added analyses. One important purpose of data cleaning is to detect and remove outliers while retaining ‘good’ data. In view of this concept, data cleaning is different from data filtering. Filtering refers to estimating the current point based upon past points. It changes the data structure by reducing not only the influence of outliers but also data variations, which may lose original data information [11]. The common GPS trajectory processing techniques such as mean filter and median filter [12] mainly focus on filtering rather than cleaning. This paper aims to clean the trajectories, i.e., detect and remove outliers.

The majority of outlier detection methods for time-dependent data are either non-model-based or model-based, depending on whether a model is needed. Non-model-based method defines a specific measure without assuming the underlying data model. Some data are then deemed as outliers if the measured values are over a predefined threshold. Specific measures in GPS trajectory often use speed, acceleration, angle change, etc. [13,14,15,16]. Such non-model-based methods in GPS trajectories can be attributed as ‘threshold methods’. Among reported studies of these methods, thresholding speed is probably the most popular one. When the speed estimated at a location exceeds a given threshold, the corresponding GPS record will be removed. However, such a non-model-based method faces the following difficulties. For a variety of trajectories in a dataset, it is hard for users to set thresholds without prior knowledge of the moving states [17]. Moreover, objects may move at an irregular speed and in varying directions, which makes it more difficult to choose the thresholds Threshold method is ineffective to detect less obvious or small outliers that influence location-critical applications such as map matching [18]. The same may occur for trajectories with a low-sample-rate. All of them may influence the performance of the threshold method for outlier detection in trajectories.

Model-based method scores potential outliers based on the degree they deviate from an ordinary pattern using a certain inherent statistical index. Generally, the first step of outlier detection for time-dependent data is to fit a model to the data and then examine its residuals [19,20]. Model-based method describes the data by the model and detects outliers according to the model, which, to a certain extent, avoids the difficulties of setting thresholds. Therefore, it is suitable for automated outlier detection. The model-based method is popular in a wide range of fields such as industrial quality control [11], network anomaly identification [21], and traffic flow investigation [22]. A traditional approach in GPS trajectory outlier detection is a Kalman-based method [23]. However, a major difficulty using this method for GPS trajectories arises from modeling the trajectories in practice. Not only because a trajectory could be quite irregular, but because various trajectories in a dataset may belong to vastly different classes of curves, which makes the modeling even more difficult [24]. As a result, although the model-based method is potentially appealing for outlier detection in GPS trajectories, its research is quite limited.

This paper proposes a new model-based approach for detecting outliers in GPS trajectories. It consists of two sequential steps. We first use a non-parameter, cubic smooth spline to extract the trend of a GPS trajectory, and then apply a time series method to model its residuals. Locations where significantly large residuals exist are considered as outliers. Compared with the existing model-based method, the new approach attempts not to model the trajectory directly but to adaptively model both the trend and residuals. It only requires setting one flexible critical value for outlier scores rather than setting various thresholds using prior knowledge. In addition, it is able to detect medium to small outliers in trajectories with low sample rates. Based on these properties of the proposed cleaning method, it is named as trend-residual dual modeling (TRDM). The remainder of the paper is organized as follows. Section 2 describes the theoretical aspects of the TRDM method. In Section 3, the performance of the TRDM method for real GPS trajectories data is analyzed and compared with the conventional threshold and Kalman-based method. Finally, concluding remarks and summary are given in Section 4.

## 2. Methods

This section introduces the TRDM method. Section 2.1 addresses the cubic smooth spline and its application in determining the trend in a GPS trajectory. Section 2.2 models the residuals of the trajectory with reference to the trend by a time series method, and finds the outliers by scoring the residuals. An automated selection of the smoothness parameter for cubic smooth spline is described in Section 2.3. Finally, we summarize an iterative algorithm for TRDM in Section 2.4.

### 2.1. Trend Modeling

**Figure 1:**
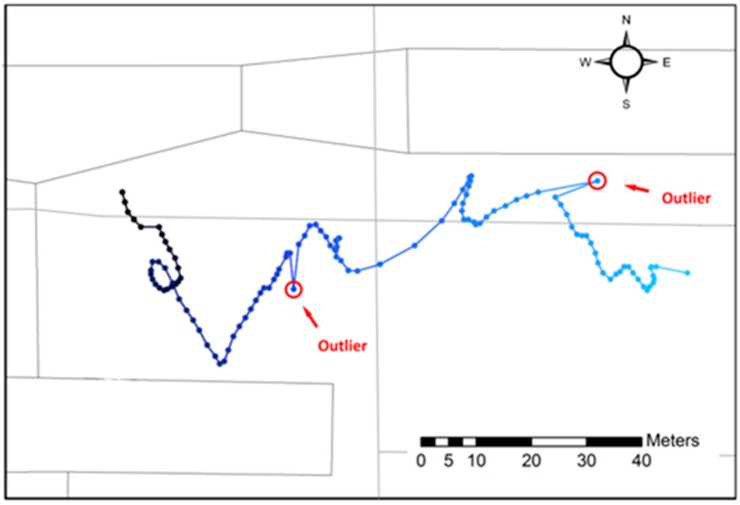
A GPS trajectory with two outliers.

This section uses cubic smooth spline to model the trend of a GPS trajectory. A GPS trajectory consists of a sequence of points with at least three measurements: time, latitude, and longitude. As an example, Figure 1 shows a trajectory of a walking person where two outliers occurred.

As a function of time, the two coordinate components of the trajectory can be expressed as below

(1) $$\begin{array}{l} Trj_{lat}=\{(t_{i},lat_{i}),1\leq i\leq n\}\\ Trj_{lon}=\{(t_{i},lon_{i}),1\leq i\leq n\} \end{array}$$

where $t_{i}$, $lat_{i}$, and $lon_{i}$ are respectively the time, latitude, and longitude of the *i-*th location in the trajectory. Since the observations are recorded in order of time, we have $t_{1}<t_{2}<\cdots <t_{n}$. Intuitively, the true trajectory should not be far from the GPS observations and is expected to be smooth to a certain degree in both longitudinal and latitudinal directions. Therefore, the cubic smooth spline of nonparametric regression is appropriate for either the latitude or longitude trajectory. It is the minimizer of the following function

(2) $$S(f)=\sum_{i=1}^{n}{\left\{Y_{i}-f(t_{i})\right\}}^{2}+\lambda \int_{a}^{b}{\left\{f^{\prime \prime }(t)\right\}}^{2}dt$$

where $Y_{i}$: the observation at $t_{i}$; λ: the pre-selected positive constant known as the smoothness (tradeoff) parameter; f(t): the expected smooth curve to be determined.

Let $Y={\left(Y_{1},\cdots ,Y_{n}\right)}^{T}$ and $\hat{F}={\left(\hat{f}(t_{1}),\cdots ,\hat{f}(t_{n})\right)}^{T}$ then it can be shown [25]

(3) $$\hat{F}={\left(I+\lambda K\right)}^{-1}Y$$

where I: the identity matrix; K: a non-negative definite band matrix determined by $t_{1},\cdots ,t_{n}$, the detail calculation refers to [25].

**Figure 2:**
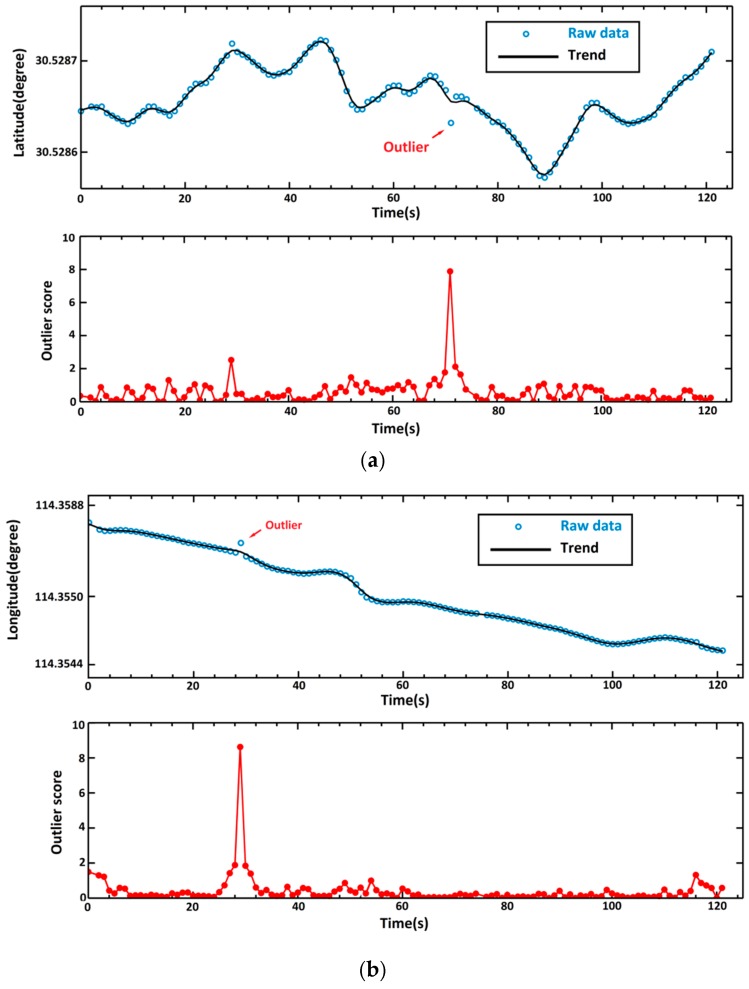
Determined trend of the trajectory (shown in Figure 1) and the outlier score for each observation in (**a**) latitude direction and (**b**) longitude direction.

The solution $\hat{f}(t)$ is a cubic spline with knots at $\hat{f}(t_{1}),\cdots ,\hat{f}(t_{n})$. It is an optimal trade-off between the goodness of fit to the data and certain smoothing requirements determined by λ. Equation (3) is essentially a penalized least squares regression. Figure 2 shows the determined trend in longitude-trajectory and latitude-trajectory for the trajectory in Figure 1. The selection of the smoothness parameter λ is crucial for the solution of $\min_{f}S(f)$ and an adaptive selection method will be introduced in Section 2.3.

### 2.2. Outlier Detection from Residuals

Once the trend is determined, outliers will be detected by modeling its residuals with reference to the observations, i.e., the GPS measurements. This is carried out with a time series method. Let $Z_{i}$ denote the original residuals, i.e.,

(4) $$Z_{i}=Y_{i}-\hat{f}(t_{i}),\;i=1,\cdots ,n$$

According to Fox [26], the residuals $Z_{i}$ contaminated by an outlier at a time stamp T can be represented by additive outlier (AO) model and innovation outlier (IO) model:

(5) $$\mathrm{AO}:\;Z_{i}=\omega I_{T}(i)+X_{i}$$

(6) $$\mathrm{IO}:\;Z_{i}=\{\begin{array}{cc} X_{i}+\omega \alpha_{i-T} & i\geq T\\ X_{i} & otherwise \end{array}$$

where ω: the magnitude of the outlier; $I_{T}\left(t\right)$: the indicator function, i.e., $I_{T}\left(t\right)=1$ if and only if t=T; $X_{i}$: the background process of outlier-free residuals; $\alpha_{i}$: the parameters of $X_{i}$’s infinite moving average representation [27].

The unobserved background process $X_{i}$ is supposed to follow the auto-regressive and moving average (ARMA) model:

(7) $$\Phi \left(B\right)X_{i}=\Theta \left(B\right)\varepsilon_{i}$$

In this case, $\alpha_{i},i\geq 0$ satisfies $\frac{\Theta (B)}{\Phi (B)}=\sum_{j=0}^{\infty }\alpha_{j}B^{j}$. For further investigation of outliers in practice, the parameters of the ARMA model of $X_{i}$ should first be estimated by the observed residuals $Z_{i}$ using robust methods, e.g., the Extended Sample Autocorrelation Function (ESACF) [28], Durbin method [29] or the methods in [27].

To score outliers in time series, let $\pi (B)=\frac{\Phi (B)}{\Theta (B)}=1-\sum_{j=1}^{\infty }\pi_{j}B^{j}$. From the single outlier models (5) and (6), we can find the least squares estimation of the magnitude of the outlier

(8) $${\hat{\omega }}_{\mathrm{AO}}(T)=\rho^{2}(1-\pi_{1}F-\ldots -\pi_{n-T}F^{n-T})\frac{\Phi (B)}{\Theta (B)}X_{T}$$

(9) $${\hat{\omega }}_{\mathrm{IO}}(T)=\frac{\Phi (B)}{\Theta (B)}X_{T}$$

where $\rho^{2}={\left(1+\sum_{j=1}^{n-T}{\pi_{j}}^{2}\right)}^{-1}$, F is the forward shift operator, i.e., $Fe_{T}=e_{T+1}$. Since $\frac{\Phi (B)}{\Theta (B)}X_{T}$ follows a normal distribution [27] with zero mean and $\mathrm{var}(\omega_{\mathrm{AO}}(T))=\rho^{2}\sigma^{2}$, $\mathrm{var}({\hat{\omega }}_{\mathrm{IO}}(T))=\sigma^{2}$, the two estimators

(10) $$\eta_{\mathrm{AO}}(T)=\frac{{\hat{\omega }}_{\mathrm{AO}}(T)}{\rho \sigma }$$

(11) $$\eta_{\mathrm{IO}}(T)=\frac{{\hat{\omega }}_{\mathrm{IO}}(T)}{\sigma }$$

both follow standard normal distribution, where $\sigma^{2}$ is the variance of Gaussian white noise $\left\{\varepsilon_{i}\right\}$. Then, by comparing the two estimators with a predefined critical value $C_{r}$, one can determine the existence of outlier at time $t_{T}$. As $\eta_{\mathrm{AO}}(T)$ and $\eta_{\mathrm{IO}}(T)$ both follow the standard normal distribution, the critical value $C_{r}$ is in fact the ratio between the magnitude of the residual’s outlier and its standard deviation, which in general is selected as 3, 3.5, and 4, respectively for high, moderate, low sensitivities to outliers in the literature [30].

In practice, we do not know how many outliers there are in a trajectory and what their time stamps are. To address this problem, Chang and Tiao [30] proposed an iteration approach. It checks the magnitudes of the two estimators at all points and then detects the most obvious outlier one at a time. Based on this idea, we define the outlier score of a point by

(12) $$\eta (i)=\max(|\eta_{\mathrm{AO}}(i)|,|\eta_{\mathrm{IO}}(i)|),\;1\leq i\leq n$$

and regard the largest one, when larger than $C_{r}$, as the most obvious outlier.

Figure 2 shows the results of trend-residual dual modeling and outlier detection for the trajectory shown in Figure 1. This trajectory is for a time period of 122 s and consists of 120 GPS recorded locations. The raw GPS recordings are shown in circles, while the trend is in solid dark lines. As depicted, the trend is a smooth curve determined by cubic smooth spline. There exist residuals between each GPS recording and the corresponding trend locations. Such residuals are then scored by using Equation (12) to identify the potential outliers. At the end, the GPS observations with outlier scores above the predefined critical value $C_{r}$ are considered as outliers.

The predefined critical value $C_{r}$ influences the cleaning results with TRDM. Generally, the smaller the value, the stronger the outlier detection ability. But at the same time, it has a greater risk to wrongly detect ‘good’ data as outliers. The effects of different critical values will be further discussed in Section 3.3 through experiments. It will be shown that TRDM is able to have a high outlier detection capability while maintaining a relatively small risk to wrongly clean the ‘good’ data.

### 2.3. Selection of the Smoothness Parameter λ

Determining the smoothness parameter λ in Equation 2 is a key step to achieve an optimal outlier detection since it actually balances the smoothness and the allowed sudden change of a trajectory. To this end, we propose a criterion that combines the generalized cross-validation (GCV) and the corrected Akaike information criterion (AIC_c_). The GCV approach is a modification of cross-validation in which the deleted residuals at points with large values are down-weighted [25]. Correcting the finite sample bias of AIC, AIC_c_ is an improved version of AIC proposed by Hurvich et al. [31]. The corresponding criteria of the two methods are:

(13) $$\mathrm{GCV}(\lambda )=\frac{1}{n}\frac{\sum_{i=1}^{n}{\left\{Y_{i}-\hat{f}(t_{i})\right\}}^{2}}{{\left\{1-n^{-1}tr(A)\right\}}^{2}}$$

(14) $${\mathrm{AIC}}_{C}(\lambda )=\log\frac{{\left\|\left(A-I\right)Y\right\|}^{2}}{n}+\frac{2\{tr(A)+1\}}{n-tr(A)-2}+1$$

where $A={\left(I+\lambda K\right)}^{-1}$ is called the ‘hat’ matrix. Technically, λ should be chosen so that Equations (13) and (14) are minimized. For our application, we suggest the final smoothness parameter as

(15) $$\lambda =\max(\lambda_{\mathrm{GCV}},\lambda_{{\mathrm{AIC}}_{C}})$$

where $\lambda_{\mathrm{GCV}}$ and $\lambda_{{\mathrm{AIC}}_{C}}$ are the maximal values respectively making GCV(λ) and ${\mathrm{AIC}}_{C}(\lambda )$ to be local minimum. Such a selection can be justified as below. The existence of GPS outliers is inconsistent with the assumption of Gaussian noise in cubic smooth spline. Larger smoothness parameters will mitigate potential influence of outliers and yield a smoother trend. In practice, since similar values of the smoothness parameters generate almost the same trends, we determine $\lambda_{\mathrm{GCV}}$ and $\lambda_{{\mathrm{AIC}}_{C}}$ based on a limited number of $\lambda \in \{5\times 10^{i-5},\;i=1,2,\ldots 10\}$.

### 2.4. Solution Procedure

In summary, the proposed TRDM method consists of the following steps:

1. Consider a trajectory data sequence $\left\{(t_{i},Y_{i}),\;i=1,2,\ldots ,n_{Y}\right\}$, where $n_{Y}$ is the number of GPS points.
2. Use the cubic smooth spline to extract the trend within data and obtain residuals.
  (a) Set the smoothness parameter λ by (15).
  (b) Estimate $\hat{f}(t_{i}),i=1,2,\ldots ,n_{Y}$ i.e., the value of data trend at $t_{i}$ by (3).
  (c) Calculate the residuals between the observations and the trend $Z_{i}=Y_{i}-\hat{f}(t_{i})$ for $i=1,2,\ldots ,n_{Y}$.
3. Use the time series method to model residuals and score outliers for every observation.
  (a) Model the ARMA (p,q) for outlier-free time series ${\left\{X_{i}\right\}}_{i=1}^{n_{Y}}$ of residuals ${\left\{Z_{i}\right\}}_{i=1}^{n_{Y}}$ by the ESACF [28] and Durbin method [29].
  (b) Calculate outlier score $\eta (i),\;i=1,2\ldots ,n_{Y}$ for each point by (12).
4. If $\max_{1\leq i\leq n_{Y}}\{\eta \left(i\right)\}>C_{r}$, where $C_{r}$ is a predetermined critical value (3, 3.5 or 4), remove the point $\left(t_{i_{0}},Y_{i_{0}}\right)$, where $\eta (i_{0})=\max_{1\leq i\leq n_{Y}}\{\eta (i)\}$.
5. Let the cleaned data be the new data sequence. Note that the number of the current data sequence $\left\{(t_{i},Y_{i}),\;i=1,2,\ldots n_{Y}\right\}$ is one point fewer than the previous data sequence. Go to step 2 until $\max_{1\leq i\leq n_{Y}}\{\eta (i)\}\leq C_{r}$.

A few notes need to be made for practical implementation of TRDM. Since a GPS trajectory is decomposed to longitudinal and latitudinal directions, the above procedure needs to run for longitudinal and latitudinal directions separately. The final cleaned trajectory is the records retained in both directions. Besides, for a long GPS trajectory, one may divide the trajectory into multiple segments. We suggest that one segment is at least 100 points to ensure correctly identifying time series model of residuals [32,33].

## 3. Experiments and Evaluation

This section presents the experimental results on two real datasets, one trajectory from a vehicle and another on foot, to demonstrate the performance of the TRDM method. The last part evaluates the performance of the TRDM method by simulating different outliers and using 10 RTK GPS trajectories as ground truth.

We compare TRDM with a traditional two-stage method. In this method, a popular threshold method that considers both velocity (V) and acceleration (A) is used, and then a Kalman-smoother based cleaner is further applied to remove outliers.

For the VA threshold method, two sets of thresholds are used. VA1 has a velocity and acceleration limit of 22 m/s and 10 m/s^2^, respectively, where the velocity limit is chosen for city zones and the acceleration limit is recommended by Chen [17]. The second threshold VA2 is set to the maximal velocity and maximal acceleration of the ground truth trajectory. VA1 threshold method is often used to clean outliers in practice. In the following examples, we compare the VA1 threshold method and TRDM method with critical value for the low-cost vehicle trajectory and walking trajectories. Since the VA2 threshold method is difficult to use in practice, it will only participate in the simulation study in Section 3.3.

The Kalman-smoother based cleaner (KSC) is a modified version of the standard Kalman smoother. KSC removes a point if it is more than certain times the standard deviation rather than smoothing every point as its earlier version does [23]. In this study, we use three times the standard deviation for comparison. To implement the KSC method, a process model for the trajectory is required. Our study uses the popular near-constant velocity model, which assumes the object moves at a constant velocity in a short time [12]. The variation parameters of KSC is estimated by Sage-Husa method [34]. KSC is a fair comparison method as both KSC and TRDM are model-based and offline, i.e., cleaning outliers based on all measurements of the trajectory [35].

### 3.1. Vehicle Trajectory

**Figure 3:**
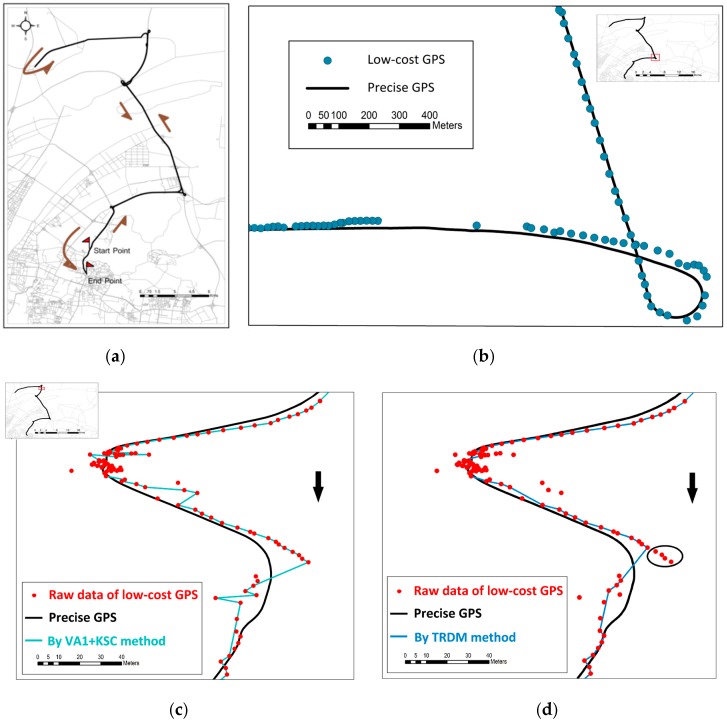
A vehicle route and its outlier detection outcomes. (**a**) Driving route; (**b**) A sample section of the route where the records of low-cost GPS fit well with the precise GPS; (**c**) Cleaned problematic trajectory segment using the VA1 + KSC method; (**d**) Cleaned problematic trajectory segment using the TRDM method.

A real example of a vehicle trajectory is used to illustrate the capability of TRDM in detecting unapparent outliers. For evaluation purposes, the trajectory was recorded by a low-cost GPS receiver and a precise GPS receiver, both aboard a Chevrolet Captiva. The low-cost GPS receiver was an Android Phone (Samsung S4 with Android version 5.0), recording at a rate of 1 Hz by using a home-made application. A precise Trimble R7 GPS receiver, configured to use RTK corrections, was employed to record the path at a rate of 2 Hz. The entire route was about 90 km (Figure 3a).

By comparing the two trajectories point-to-point in terms of time recorded by the low cost GPS and the professional Trimble GPS with RTK correction, it is found that the mean of distance errors between the two trajectories is 15.97 m, and the root mean square error (RMSE) is 13.48 m. With the TRDM method, the mean and RMSE of distance errors are reduced to 15.17 m and 12.70 m respectively. Such marginal improvement is because the low-cost GPS trajectory overall did not deviate greatly from the RTK GPS trajectory. Figure 3b shows an example section of the route where the low-cost GPS returns a trajectory quite close to the RTK GPS trajectory.

However, the TRDM method shows its contribution in a complex, curving segment as shown in Figure 3c,d. The low-cost GPS recorded many erroneous points possibly due to the low driving speed (30 km/h) in this curved segment. Figure 3c shows many remaining outliers after VA1 + KSC method, since the velocity change is relatively small at this route segment. As a result, the KSC is less effective to identify the outliers due to its assumption of near-constant velocity. In contrast, the TRDM method (Figure 3d) is able to remove many such small outliers (typically the ones in the circle). This is because the introduction of the trend in TRDM enables adaptive capture of the structure of the trajectory and detect points that are inconsistent with their neighboring ones.

**Figure 4:**
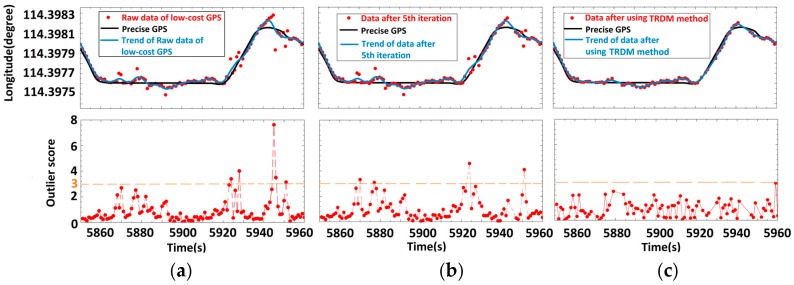
Iterations of the TRDM method in longitude for the problematic segment of Figure 3d. (**a**) Raw data; (**b**) the fifth iteration; (**c**) cleaned data.

The mechanism of the TRDM’s satisfactory performance can be illustrated by examining the longitude direction of this problematic segment. Figure 4a shows the trend of raw data extracted by cubic smooth spline. The trend fluctuates around the precise GPS, and the points deviating from precise GPS significantly are also deviating from the trend further compared with other ‘normal’ points. A great deviation from the trend contributes a high outlier score. Notice that the trend near 5940s balances the points before and after in longitude direction, which helps us detect the deviations. However, some erroneous points such as the ones near 5870s and 5875s have outlier scores lower than $C_{r}=3$ at the start. After the fifth iteration (Figure 4b), the outlier scores of remaining points increase in certain degree (e.g., erroneous points near 5870s and 5875s) and have outlier scores higher than $C_{r}=3$. That is a more obvious outlier and it may suppress the outlier scores of other erroneous points. Therefore, detecting and removing all outliers at one time is difficult. This is one of the reasons we iterate to remove outliers one at a time. Finally, we stop the iteration until all the outlier scores are lower than 3. From Figure 4c, we find that the remaining points are all close enough to the precise GPS, and the trend is more similar with the one comparing with the raw data.

### 3.2. Walker Trajectories

**Figure 5:**
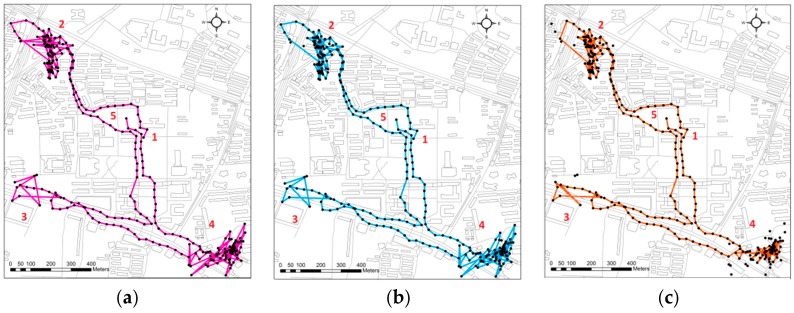
A volunteer walking trajectory, black points are the raw data. (**a**) Original trajectory; (**b**) “Cleaned” trajectory by VA1 + KSC method; (**c**) “Cleaned” trajectory by TRDM method.

We now discuss the performance of TRDM in more complex low-sample-rate walker trajectories. A walking trajectory (Figure 5a) was collected by a volunteer with a smartphone under Android OS in an urban setup at a sample rate 5 Hz. Due to the obstructions from buildings and the weather at that time or other unknown reasons, there were many missing points. The actual mean sample rate was 40 s. The route was from Place 1 to Place 5, and at Places 2 and 4 the volunteer wandered in a small range, causing a large deviation from the main trajectory. The VA1 + KSC method shown in Figure 5b has little ability to detect and remove the deviations near Places 2 and 4, while the TRDM cleans most of deviations, especially at Place 4. The cleaned trajectory in Figure 5c converges closely at Place 4 and all the points wrongly cross the streets are excluded. This is helpful for understanding the volunteer’s movement more clearly.

**Figure 6:**
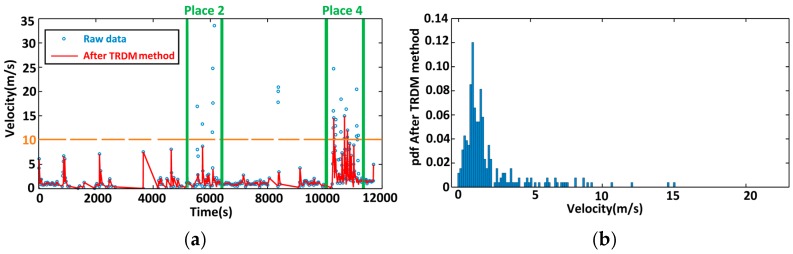
Walking velocity of the volunteer after TRDM method. (**a**) Velocity before and after cleaning; (**b**) Velocity probability distribution after TRDM method.

Figure 6 shows the velocity of the volunteer after using the TRDM method. At Places 2 and 4, the original velocities of many points exceed 10 m/s which are beyond walking speed limit, and the variation is large. After the TRDM method, most of velocities are less than 10 m/s. What is more, the standard deviation and the 95th-pencentile range are computed and shown in Table 1, in which the standard deviation and 95th-pecentile are reduced by 42.89% and 40.65%, respectively. It implies that, after applying the TRDM method, the velocity is relatively stable in a more reasonable range of common sense than before.

**Figure 7:**
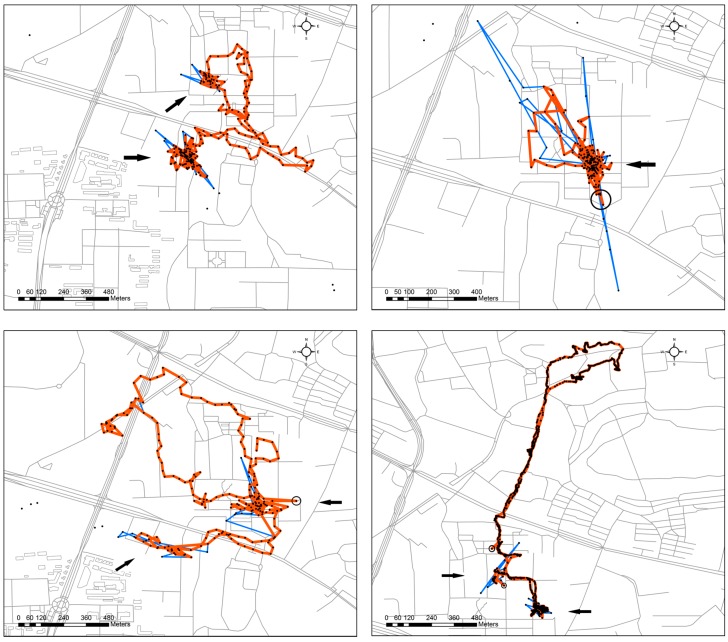
Four volunteers’ trajectories and their VA1 + KSC (blue) and TRDM (red) cleaning results. The black points are the raw data. The arrows show the detected wandering locations. The black circles show suspected outliers which still exist after TRDM.

Finally, Figure 7 shows the TRDM results of the other four volunteers’ trajectories with low sample rates collected with smartphones under Android OS. The arrows show the wandering places of the volunteers. After using the TRDM method, many large deviations are removed. These wandering places then become much apparent, although a few suspected outliers (e.g., those in black circles) still exist. The possible reason for the remaining outliers after the TRDM method is that the suspected outliers influence the trends, and then the remaining outliers do not deviate from the trends obviously enough. Therefore, the time series method is unable to detect them.

### 3.3. Performance Evaluation

This section evaluates the performance for the TRDM method by adding a range of outliers to a set of precise RTK GPS trajectories. For comparative purpose, results from the VA1 + KSC and VA2 + KSC methods are provided. Finally, the time consumption of TRDM and VA1 + KSC is discussed.

**Figure 8:**
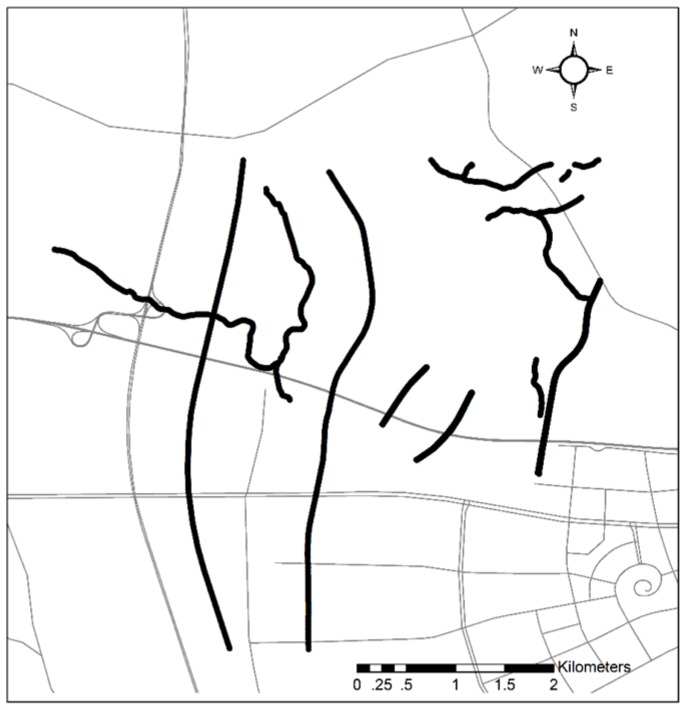
Ten RTK GPS vehicle trajectories overlaid atop road map.

Ten precise GPS trajectories are used as ground truth in this study (Figure 8). They were collected by a Leador Spatial mobile mapping system equipped with a precise Trimble R8 GPS receiver with RTK corrections. The sample frequency was 1 Hz for most of the time, whereas there was a small part of data collected at a lower rate. We define small, medium, and large three outlier classes that respectively have a magnitude of errors of 0.00015 degree (about 15.7 m), 0.0004 degree (about 41.9 m), and 0.001 degree (about 104.7 m). We create four groups of simulated datasets by contaminating 10% of every trajectory respectively with small, medium, large outliers, and a mixture of the above three. Every contamination version is simulated 100 times for each trajectory. Outliers are added randomly to the original trajectories.

We use two metrics for evaluation: FP rate (i.e., false positive or type Ι error) and FN rate (i.e., false negative or type Π error). FP rate is the rate of wrongly detecting normal observations as outliers, while FN rate is the rate of undetected outliers.

**Figure 9:**
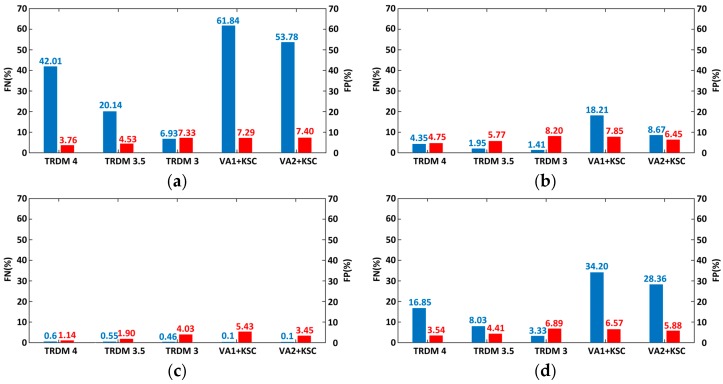
False negative (blue) and false positive (red) rates for the TRDM and VA + KSC methods. The contamination modes are: (**a**) 10% small outliers; (**b**) 10% medium outliers; (**c**) 10% large outliers; (**d**) mixture outliers.

Shown in Figure 9 are the statistical averages for all 4000 simulations from TRDM and VA + KSC methods under different contamination modes. The VA + KSC methods could detect almost all of the large deviations, but VA1 + KSC could not detect as many as VA2 + KSC does when the outlier magnitudes are medium. Notice that both VA + KSC methods perform poorly for small outliers and outliers of mixture magnitudes. Since we often do not know the actual maximum speed and acceleration, the adoption of VA1 + KSC would only be able to handle large outliers and some medium outliers in practice. On the other hand, the TRDM method never performs worse than the VA + KSC methods (though all working well for large outliers), especially in detecting small and mixture outliers. Moreover, the TRDM method can detect most of the outliers no matter which critical values are set. However, the detection efficiencies of TRDM with critical value $C_{r}$ = 3.5 and 4 performed slightly poor for small and mixed contaminated outliers, though they still correctly remove much more outliers than the VA + KSC methods.

With the decrease of the critical values from $C_{r}$ = 4 to 3.5 and 3, the false negative rates of TRDM decrease while false positive rates increase. It implies that TRDM with a smaller critical value filters out more outliers and more normal data at the same time which agreed with general knowledge. In addition, the false positive rates of TRDM with the smallest critical value of 3 are just a little higher than VA1 + KSC method. It shows that the TRDM method retains most of normal data as the popular VA1 + KSC method. Among the four contamination modes, the mixture outlier mode is the most similar one to the reality. Therefore, loosely speaking, the most aggressive TRDM method (with critical value $C_{r}=3$) can reach an average FN rate 9.27 (34.2/3.33−1) times lower than the VA1 + KSC method, while the FP rate is only 0.05 (6.89/6.57−1) times higher, as shown in Figure 9d.

**Figure 10:**
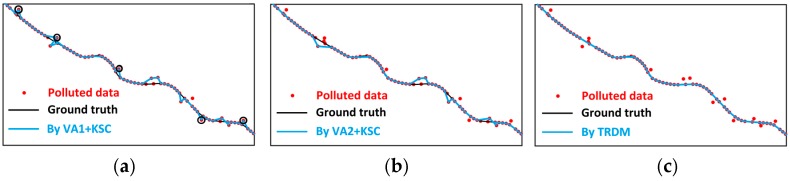
Comparison of outlier detection by VA + KSC and TRDM methods. (**a**) VA1 + KSC; (**b**) VA2 + KSC; (**c**) TRDM method.

Taking the mixed outliers as an example, Figure 10 shows a snapshot of outlier detection and removing by the VA + KSC and TRDM methods. Thanks to appropriate thresholds, the VA2 + KSC method is able to detect five additional outliers (as shown by black circles in Figure 10a) than the VA1 + KSC method. It is, however, unable to clean less obvious outliers (see Figure 10b). Figure 10c shows that the TRDM method is sensitive in detecting medium and small outliers and removes all outliers in this segment where the VA2 + KSC method fails. This is because that the TRDM method removes the points against the trend rather than some pre-determined features and model, which makes it more effective. As a result, the cleaned trajectory segment using the TRDM method satisfactorily coincides with the ground truth.

In order to determine which critical value ($C_{r}$ = 4, 3.5, or 3) to use in practice, we suggest users to choose a lower one (e.g., $C_{r}=3$) since a smaller critical value tends to be more restrictive in keeping a potential outlier and only have a small risk of cleaning ‘good’ data. However, users can adjust this value to meet their own needs. For example, users can choose a larger critical value to retain more points if the number of records in the trajectory is relatively small, or they use robust analysis tools to reduce the influence of small outliers.

Finally, we address the time consumption of TRDM ($C_{r}=3$) and VA1+KSC. Since the two methods generally remove different numbers of points in a trajectory, the time cost of removing one point will be discussed. The scenario is based on 10 precise trajectories under the mixture outlier contamination mode with each having 100 simulations. With Inter Core i7-4790 CPU, the average times for removing one point for TRDM and VA1 + KSC are respectively 0.68 s and 0.10 s. This shows that TRDM is more time-consuming than VA1 + KSC since TRDM involves matrix inversion which is computationally costly. However, considering TRDM is an offline cleaning method and performs much better than VA1 + KSC, high-performance computing environments will be helpful for TRDM cleaning trajectories. Furthermore, there is a great potential to improve the TRDM implementation since our coding at this time is with Matlab without engineering optimization.

## 4. Conclusions

This paper proposed a model-based outlier detection approach to clean trajectories recorded by low-cost GPS. The main procedure of TRDM involved trend extraction and outlier score computation through residuals. To model the trend, we applied the cubic smooth spline to the longitude trajectory and latitude trajectory separately. The residuals of GPS trajectories with reference to the trends were then evaluated by the time series method to determine potential outliers one at a time. All outliers were found through iteration to reach a reliable, outlier-free trajectory.

Unlike other model-based methods such as KSC which face difficulty when modeling GPS trajectories, TRDM extracts the trend of a trajectory adaptively and then models the residuals. As a result, TRDM is able to clean trajectories regardless what kinds of the curves they actually belong to. Compared with various common non-model-based threshold methods, TRDM focuses on removing outliers against the intrinsic consistency of the GPS trajectory rather than some predetermined simple thresholds. Moreover, adaptive parameter estimations are introduced in TRDM and only one critical value $C_{r}$ for outlier scores needs to be set without much difficulty and prior knowledge.

Our experiments showed that TRDM could be applied to various complex GPS trajectories and it performs much better than popular velocity or acceleration threshold methods, especially when small and medium outliers exist. TRDM can yield an average false positive rate 9.27 times better than the conventional VA+KSC method, whereas its false negative is only 0.05 times higher than the VA + KSC method. More importantly, TRDM is able to detect outliers in moving objects that behave ‘normal’ in velocity or acceleration and ‘wander’ in a stop-and-go mode even if the recording time intervals are long. The resultant outlier-free trajectories are all closer to the actual trajectories and accord with common sense.

However, there are still some limitations of TRDM method which need to be improved in the future work. First, the window size in this paper is chosen by authors’ experiences to balance the speed variation and sufficient number of points to model the time series. A more reasonable adaptive selection method is desired. One may use some trajectory partition approaches (see [36] and references therein) to preprocess trajectories and then apply TRDM to clean the sub-trajectories. Second, the TRDM method may still fail to identify relatively small outliers. This problem may be solved if a digital vector map is incorporated into the TRDM method. Finally, the processing speed of TRDM is slow at this time, since the matrix inversion in trend-residual modeling is computationally costly. It may cause limitations in some time-critical, dynamic cleaning applications.

## Figures and Tables

**Table 1:** Statistical parameters of velocity of comparative results.

	Standard Deviation	95th-Pecentile
VA1 + KSC	4.01	12.03
TRDM	2.29	7.14
